# Metabolomic Analyses Reveal Extensive Progenitor Cell Deficiencies in a Mouse Model of Duchenne Muscular Dystrophy

**DOI:** 10.3390/metabo8040061

**Published:** 2018-10-03

**Authors:** Josiane Joseph, Dong Seong Cho, Jason D. Doles

**Affiliations:** Department of Biochemistry and Molecular Biology, Mayo Clinic, Rochester, MN 55905, USA; joseph.josiane@mayo.edu (J.J.); cho.dong@mayo.edu (D.S.C.)

**Keywords:** metabolomics, Duchenne muscular dystrophy, stem cells, skeletal muscle, adipose tissue

## Abstract

Duchenne muscular dystrophy (DMD) is a musculoskeletal disorder that causes severe morbidity and reduced lifespan. Individuals with DMD have an X-linked mutation that impairs their ability to produce functional dystrophin protein in muscle. No cure exists for this disease and the few therapies that are available do not dramatically delay disease progression. Thus, there is a need to better understand the mechanisms underlying DMD which may ultimately lead to improved treatment options. The muscular dystrophy (MDX) mouse model is frequently used to explore DMD disease traits. Though some studies of metabolism in dystrophic mice exist, few have characterized metabolic profiles of supporting cells in the diseased environment. Using nontargeted metabolomics we characterized metabolic alterations in muscle satellite cells (SCs) and serum of MDX mice. Additionally, live-cell imaging revealed MDX-derived adipose progenitor cell (APC) defects. Finally, metabolomic studies revealed a striking elevation of acylcarnitines in MDX APCs, which we show can inhibit APC proliferation. Together, these studies highlight widespread metabolic alterations in multiple progenitor cell types and serum from MDX mice and implicate dystrophy-associated metabolite imbalances in APCs as a potential contributor to adipose tissue disequilibrium in DMD.

## 1. Introduction

Duchenne muscular dystrophy (DMD) is an X-linked neuromuscular disease caused by mutations in the gene responsible for dystrophin production. Dystrophin is expressed primarily in muscle; however, several isotypes are also known to be moderately expressed in subsets of cells in the brain [1]. Patients with DMD typically experience progressive loss of skeletal muscle; leaving them symptomatic by age 5, wheelchair bound by age 12, and often deceased within the second decade of life [2]. Approximately 1 out of 7250 males age 5 to 24 years in the United States [3] are affected by DMD, as well as the milder dystrophinopathy, Becker muscular dystrophy. There is currently no cure or effective treatment for this genetic disorder despite decades of research using animal models with analogous pathology [4].

Since its generation in 1984 [5], the X-linked muscular dystrophy (MDX) mouse has become widely established as a valuable tool for understanding DMD. Similar to patients with muscular dystrophy, MDX mice are dystrophin deficient and display signs of cardiomyopathy and muscle fibrosis [6,7]. In contrast to the human disorder, MDX mice only exhibit slightly reduced lifespans when compared to wildtype (WT) mice [7] and the mice also retain skeletal muscle functionality throughout life. This discrepancy (or milder disease phenotype) is thought to be due to compensation by the related structural protein utrophin in mice [8,9]. 

There are few comprehensive metabolic studies of muscular dystrophy using mouse models [10] and even fewer focusing on metabolic alterations in individual cell types, such as muscle satellite cells (SCs), previously implicated in the disease [11,12]. Some studies of samples from patients and MDX mice describe subsets of metabolic alterations in skeletal [13,14,15,16] and cardiac muscle [17], brain [18], and serum [19]. However, the extent of these metabolic deficiencies within skeletal muscle and in surrounding tissues is unclear. 

Of note, little is known about metabolic alterations in dystrophic tissue progenitor cell populations, which is a key knowledge gap considering the importance of tissue progenitor cells to the lifelong maintenance of many adult organ systems. In this study we examined metabolic changes in two different tissue-specific adult stem cell compartments: skeletal muscle SCs and primary adipocyte precursors. We used nontargeted metabolomics to assess global metabolite abundance and composition in MDX cells compared to WT controls. Consistent with previously published studies, we found widespread dysregulation of many metabolic pathways in MDX serum and skeletal muscle progenitor cells. Interestingly, we observed cell autonomous defects in dystrophic adipose progenitor cell (APC) function. Metabolomic analyses of APCs revealed aberrant accumulation of fatty acid metabolism intermediates, which we show can negatively impact primary cell growth. Together, this study highlights the extent to which dystrophic skeletal muscle alters the systemic metabolic milieu, and underscores the importance of metabolic imbalances in the maintenance and function of adult tissue progenitor cells.

## 2. Results

### 2.1. Satellite Cells (SC) Isolated from Dystrophic Mice Exhibit Metabolite Imbalances

**Figure 1:**
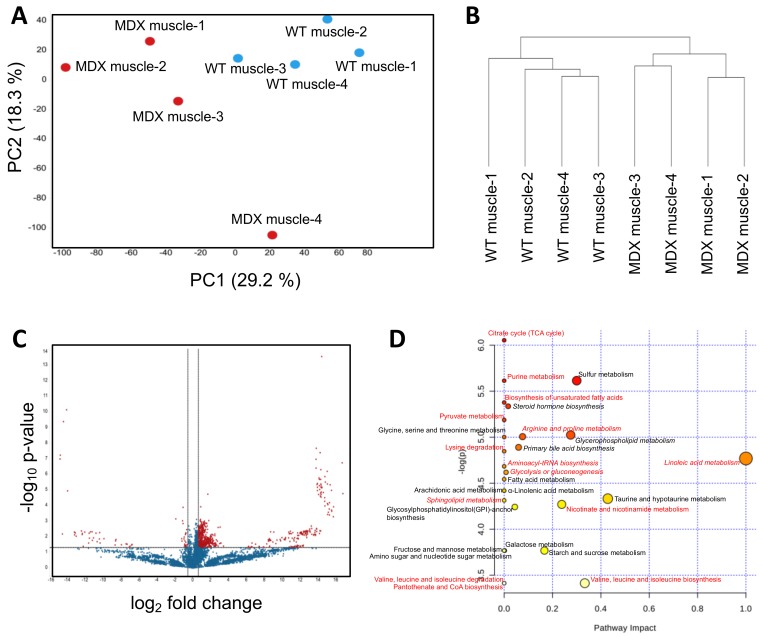
Metabolomic analysis of muscle satellite cells. (**A**) A principal component analysis (PCA) plot derived from nontargeted metabolite profiling of muscle satellite cells isolated from four WT control and four MDX mice. (**B**) A dendrogram depicting hierarchical clustering results of samples from (**A**). Clustering method: complete linkage. Distance measurement: Euclidean. (**C**) A volcano plot depicting differentially abundant metabolites (DAMs) between WT and MDX satellite cells (SCs). Horizontal and vertical dashed lines represent a threshold of *p*-value 0.05 and fold change 1.5, respectively. Seven-hundred-and-fifty-five features were identified to be significant from 11,151 features. (**D**) Pathway analysis of SC DAMs. Italics: pathways with multiple metabolites implicated. Red: metabolic pathways identified in both serum and muscle satellite cell analyses.

Accumulating evidence suggests that defects in muscle SCs contribute to the dystrophic state by limiting skeletal muscle repair and regeneration [20,21]. While many studies have examined dystrophic SC signal transduction and gene expression [22,23], opportunities to explore dystrophic SC metabolism remain. We isolated primary SCs from the hind limb muscles of WT control and dystrophic (MDX) mice using an antibody/magnetic bead-based approach that enriches for integrin α-7-positive cells (SCs) (Figure S1). We then performed nontargeted metabolomic analyses using a liquid chromatography–mass spectrometry (LC–MS) based approach (see Methods for experimental details). On the basis of intracellular metabolite differences, principal component analyses (PCA) showed clear separation of WT SCs from dystrophic SCs (Figure 1A). The first and second PCA components explained 29.1% and 18.3% of variations, respectively. Hierarchical clustering (method: complete linkage; distance measurement: Euclidean) corroborated the PCA and showed robust separation of MDX SCs from WT SCs (Figure 1B). Statistical analyses (*t*-test) revealed 755 significant differentially abundant features (metabolites) from 11,151 total features (6.7%) (Figure 1C). 

Among the top differential metabolites with annotations in the Human Metabolome Database (HMBD) were fatty acid derivatives, including several long-chain carnitine species (elaidic carnitine, linoleyl carnitine, palmitoyl-l-carnitine, and stearoyl-l-carnitine), and amino acid compounds (Table S1). MetaboAnalyst-guided pathway enrichment analysis revealed alterations in multiple metabolic pathways including linoleic acid metabolism, glycerophospholipid metabolism, taurine metabolism, branched-chain amino acid biosynthesis, and nicotinamide metabolism (Figure 1D). Together, these data support previous observations that dystrophic SCs exhibit functional metabolic deficiencies [24], particularly with respect to beta oxidation and mitochondria function. 

### 2.2. Dystrophic Mice Have Serum Metabolite Alterations

**Figure 2:**
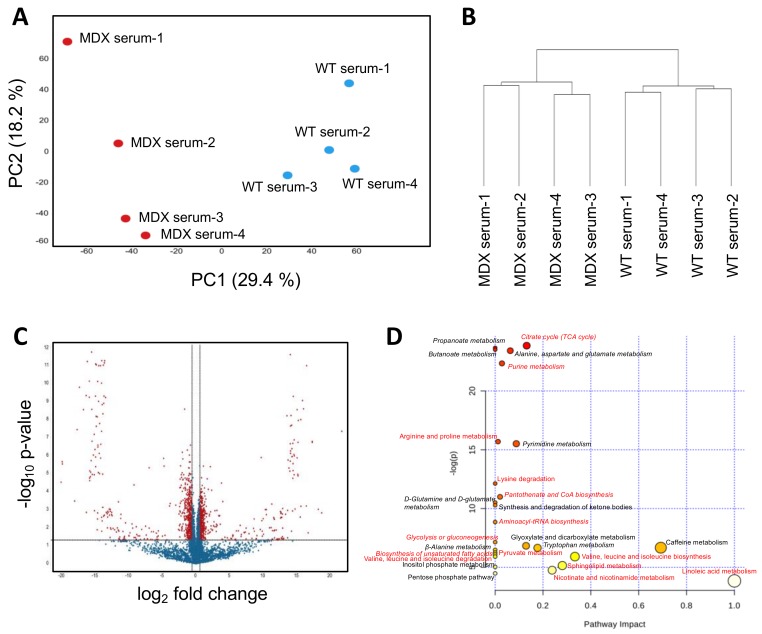
Serum metabolomic analysis. (**A**) A principal component analysis (PCA) plot derived from nontargeted metabolite profiling of serum isolated from four WT control and four MDX mice. (**B**) A dendrogram depicting hierarchical clustering results of samples from (**A**). Clustering method: complete linkage. Distance measurement: Euclidean. (**C**) A volcano plot depicting DAMs between WT and MDX serum. Horizontal and vertical dashed lines represent a threshold of *p*-value 0.05 and fold change 1.5, respectively. One-thousand-one-hundred-and-twenty-five features were identified to be significant from 9562 total features. (**D**) Pathway analysis of serum DAMs. Italics: pathways with multiple metabolites implicated. Red: metabolic pathways identified in both serum and muscle satellite cell analyses.

Prior studies examining serum metabolites from dystrophic mouse models and patients have largely consisted of targeted studies of relatively small metabolite panels. We sought to perform a controlled and unbiased assessment of the dystrophic serum metabolome using nontargeted metabolomics. PCA of WT control versus MDX mouse serum samples revealed clear separation between the two groups (Figure 2A). The first and second PCA components explained 29.4% and 18.2% of variations, respectively. Hierarchical clustering (method: complete linkage; distance measurement: Euclidean) similarly separated MDX mouse serum compared to control serum (Figure 2B). Statistical analyses (*t*-test) revealed 1125 significant differentially abundant features (metabolites) from 9562 total features (11.8%) (Figure 2C).

Among the top overrepresented metabolites in MDX serum with annotations in the Human Metabolome Database (HMBD) were purine/pyrimidine metabolites (including hypoxanthine, oxypurinol, and adenosine) and fatty acid metabolites. Metabolites selectively depleted in MDX serum included amino compounds (D-alanine, thymidine, and D-glutamate) and tricarboxylic acid (TCA) associated metabolites (including succinic acid, lactic acid, and oxoglutaric acid) (Table S2). MetaboAnalyst-guided pathway enrichment analysis confirmed alterations in multiple metabolic pathways including citrate (TCA cycle), alanine, aspartate and glutamate, and purine and pyrimidine metabolism (Figure 2D). Several metabolic pathways including glucose metabolism, amino acid (valine, leucine, isoleucine, arginine, proline, and lysine) metabolism, and unsaturated fatty acid metabolism were also identified as differentially abundant in SCs from MDX mice (see pathways labeled with red color in Figure 2D and Venn diagram in Figure S2). Together these studies (1) show that dystrophic serum exhibits marked changes in circulating metabolites, (2) confirm prior studies that report circulating amino acid deficits [25,26], and (3) highlight novel metabolic changes, such as elevated purine/pyrimidine metabolites that may represent deficiencies in skeletal nucleotide metabolism. 

### 2.3. Dystrophic Mice Exhibit Adipose Tissue Abnormalities

**Figure 3:**
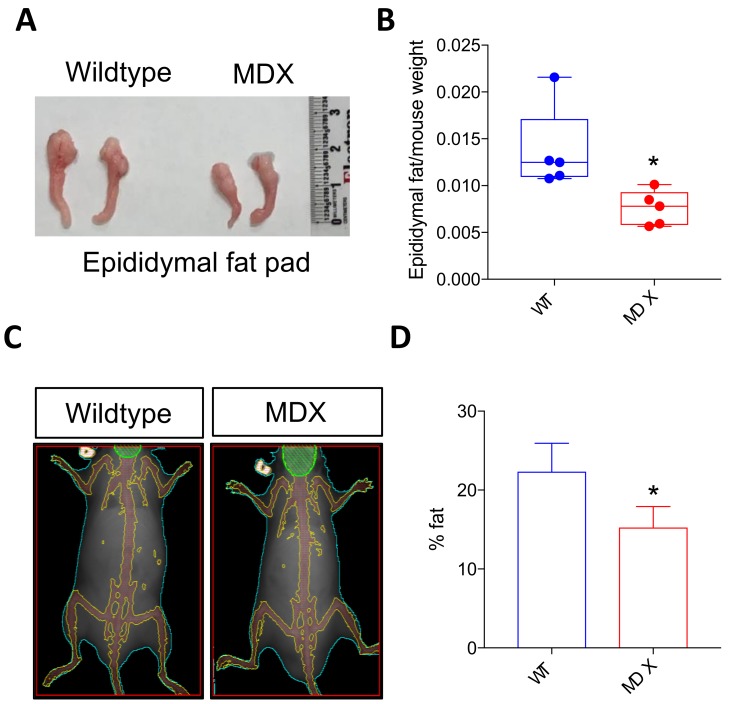
MDX mice exhibit decreased adipose tissue mass. (**A**) Images depicting WT and MDX epididymal fat pads. (**B**) A bar graph depicting epididymal fat pad weight normalized to total mouse weight. * *p* < 0.05. *n* = 5 mice in each experimental group. (**C**) Representative dual-energy X-ray absorptiometry (DEXA) images of WT and MDX mice. (**D**) Quantification of fat percentage based on DEXA image analysis. * *p* < 0.05. *n* = 4 mice in each experimental group.

DMD patients often exhibit alterations in adipose tissue, with increased incidence of obesity observed in younger DMD patients, followed by dramatic adipose tissue loss with advanced age [27,28]. Similar to human muscular dystrophy patients, MDX mice experience irregular increases in weight early in life [29]. Gross inspection of epididymal fat pads from 4 to 6 month old male mice revealed reduced fat in MDX mice (Figure 3A). Indeed, epididymal fat pad wet weights in MDX mice were statistically smaller than control fat pad weights (normalized to total body weight; *p* = 0.0224; *n* = 5 mice in each group) (Figure 3B). We then performed whole-body dual-energy X-ray absorptiometry (DEXA) to assess total body composition. The average percent body fat calculations revealed less total body fat in MDX mice compared to control mice (~22.5% fat in control mice versus ~15% total fat in MDX mice; *p* = 0.0291) (Figure 3C,D). 

**Figure 4:**
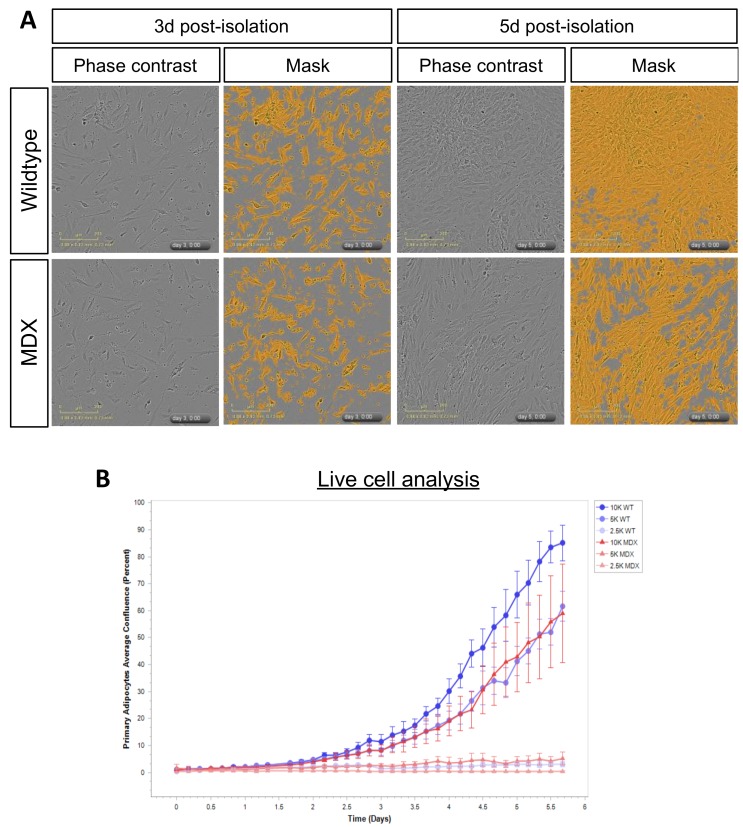
Primary adipose progenitor cells (APCs) from MDX mice exhibit in vitro expansion defects. (**A**) Representative images of APC cultures derived from WT and MDX mice at 3d and 5d post-isolation. Shown are phase contrast images and corresponding mask overlays used for proliferation quantification analyses. (**B**) Line graphs depicting proliferation curves of WT (blue curves) and MDX (red curves) APCs over a 5d time-course. Shown are proliferation curves based on initial seeding densities of 2.5 K (light coloring), 5 K (medium coloring), and 10 K (dark coloring) APCs/well of a 96-well plate.

Based on the observation that MDX mice displayed less adipose tissue mass, we next asked whether primary APCs isolated from MDX mice exhibited defects upon in vitro culture. Control or MDX adipose tissue was mechanically and enzymatically processed (see Methods), and single cell preparations enriched in APCs plated for live-cell imaging analyses. APC purity was assessed by staining enriched cells with the APC marker, Sca1 (Figure S3). Visual inspection of WT and MDX APCs did not reveal gross alterations in APC morphology (Figure 4A), however we did observe proliferation defects in MDX APCs compared to WT controls (Figure 4A,B). These differences were observed at several plating densities and were most apparent when APCs were plated at a density of 5000 cells/well (96 well plate) (Figure 4B). Together, these data confirm adipose tissue dysfunction in MDX mice and highlight persistent cell-autonomous expansion defects in MDX APCs cultured ex vivo. 

### 2.4. MDX Adipose Progenitor Cells (APCs) Have Altered Metabolite Profiles

**Figure 5:**
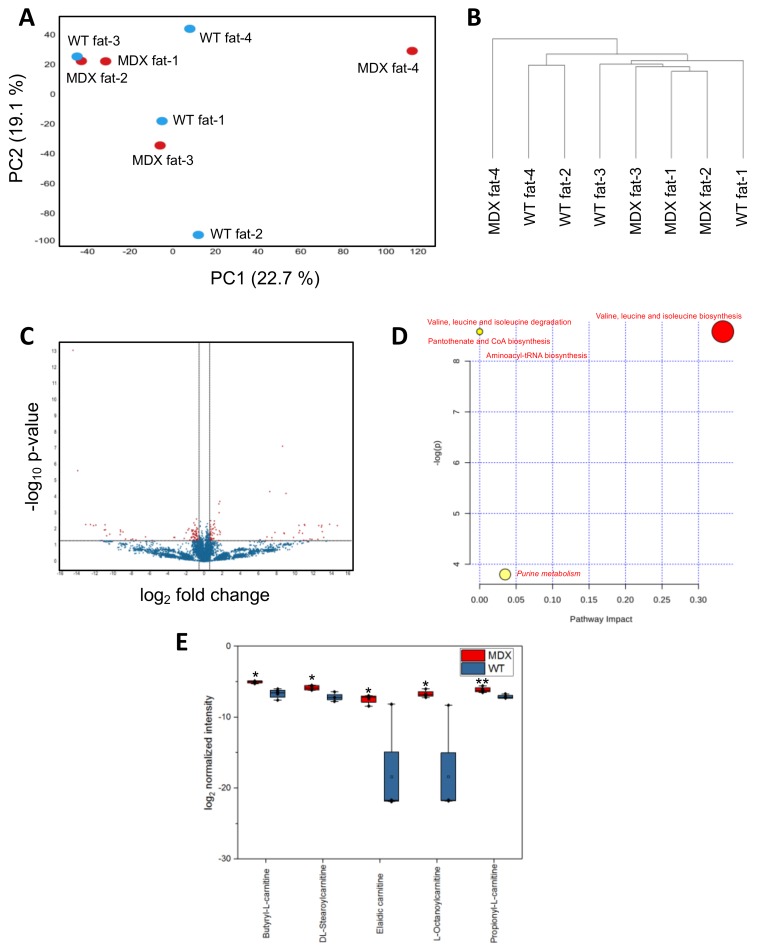
APC metabolomic analysis. (**A**) A principal component analysis (PCA) plot derived from nontargeted metabolite profiling of APCs isolated from four WT control and four MDX mice. (**B**) A dendrogram depicting hierarchical clustering results of samples from (**A**). Clustering method: complete linkage. Distance measurement: Euclidean. (**C**) A volcano plot depicting DAMs between WT and MDX APCs. Horizontal and vertical dashed lines represent a threshold of *p*-value 0.05 and fold change 1.5, respectively. One-hundred-and-fifty-three features were identified to be significant from 11,151 total features. (**D**) Pathway analysis of serum DAMs. Italics: pathways with multiple metabolites implicated. Red: metabolic pathways identified in common with both serum and muscle satellite cell analyses. (**E**) A bar graph quantifying the relative abundance of five acylcarnitine species. * *p* < 0.05, ** *p* < 0.01. *n* = 4 mice in each experimental group.

Considering observed changes in (1) serum metabolite levels, (2) adipose tissue stores, and (3) in vitro APC expansion, we next asked if MDX APCs exhibited steady state metabolite imbalances compared to WT control APCs. As in our SC and serum analyses, isolated APCs were subjected to nontargeted metabolomic analyses. PCA did not reveal clear separation of WT APCs from dystrophic APCs (Figure 5A). The first and second PCA components explained 22.7% and 19.1% of variations, respectively. Hierarchical clustering analyses were able to better distinguish WT from MDX APCs, with three of four MDX samples clustering away from WT controls (Figure 5B). Statistical analyses (*t*-test) revealed 153 significant differentially abundant features (metabolites) from 11,151 total features (1.4%) (Figure 5C). 

MetaboAnalyst-guided pathway enrichment analysis revealed alterations in valine, leucine, and isoleucine metabolism, pantothenate and CoA biosynthesis, aminoacyl-tRNA biosynthesis, and purine metabolism—all of which were also identified in SC and serum analyses (Figure 5D and Figure S2). Closer examination of the top differentially abundant metabolites in MDX APCs with HMBD annotations revealed a cluster of carnitine-containing fatty acid metabolism compounds that were upregulated in MDX samples (Figure 5E, Table S3). Interestingly, we also observed accumulation of several carnitine metabolites in dystrophic SCs (Figure S4), implicating fatty acid metabolism defects in multiple dystrophic progenitor cell populations. Together, these data show that APCs in MDX mice exhibit steady state metabolic alterations and suggest that carnitine accumulation in MDX APCs may contribute to the observed functional deficits.

### 2.5. Long-Chain Acylcarnitines Inhibit APC Expansion

**Figure 6:**
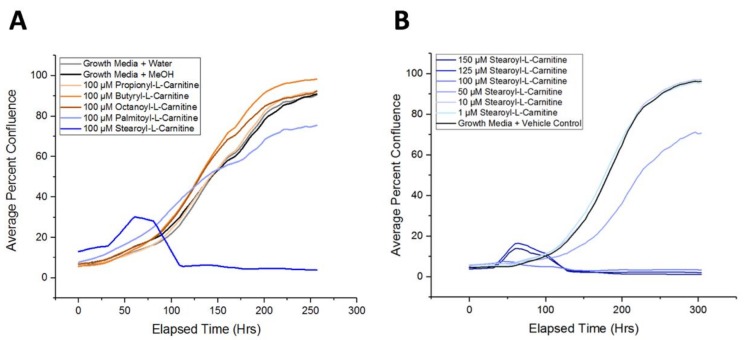
Exposure to selected acylcarnitines inhibits in vitro APC expansion. (**A**) A line graph depicting APC proliferation over a >10-day timecourse. Shown are representative proliferation traces of APCs treated with a vehicle control (water or MeOH), propionyl-l-carnitine (yellow), butyryl-l-carnitine (orange), octanoyl-l-carnitine (brown), palmitoyl-l-carnitine (light blue), or stearoyl-l-carnitine (dark blue). (**B**) A line graph depicting APC proliferation when exposed to increasing concentrations of stearoyl-l-carnitine.

Using live-cell imaging to monitor APC expansion, we next asked if individual acylcarnitines could modulate APC proliferation. We tested the acylcarnitine derivatives that were identified to be upregulated in MDX APCs (propionyl-l-carnitine, butyryl-l-carnitine, octanoyl-l-carnitine, and stearoyl-l-carnitine), as well as palmitoyl-l-carnitine, which was upregulated in MDX SCs. APCs cultured in the presence of propionyl-l-carnitine, butyryl-l-carnitine, and octanoyl-l-carnitine did not exhibit alterations in primary APC expansion over a 10-day period (Figure 6A). Supplementation with longer chain acylcarnitines (palmitoyl-l-carnitine and stearoyl-l-carnitine), however, resulted in marked impairment of APC expansion (Figure 6A). Indeed, stearoyl-l-carnitine was able to suppress APC proliferation in a dose-dependent manner (Figure 6B). Overall we show that longer chain acylcarnitines can impair APC proliferation, thus demonstrating that metabolic alterations associated with muscular dystrophy can extend beyond skeletal muscle to other non-muscle cell types. 

## 3. Discussion

A cardinal feature of DMD is metabolic dysfunction. Decades of research into specific metabolic deficiencies have implicated a host of dysregulated metabolic processes including glycogenolysis [30], glucose metabolism [31], free fatty acid and ketone body metabolism [31], acyl CoA metabolism [32], mitochondrial metabolism [33], and amino acid metabolism [34]. We performed unbiased, nontargeted metabolomic analyses on isolated murine muscle SCs and confirmed defects in many of these previously implicated metabolic pathways while also highlighting lesser known metabolites and metabolic pathways including purine metabolism and amino-acyl tRNA metabolism. These data show that metabolic alterations observed at the level of whole muscle also extend to tissue resident SCs (i.e., muscle stem cells). Whether these alterations contribute to known deficits in dystrophic SC behavior [11,20,21] would be an interesting avenue to pursue in further studies. 

A notable feature in our SC metabolomics data set was the upregulation of numerous long chain acylcarnitine (LC-AC) species. Altogether, we observed statistically significant accumulation of four LC-AC compounds: elaidic carnitine (C18), linoleyl carnitine (C18), palmitoyl-l-carnitine (C16), and DL-stearoylcarnitine. Indeed, a number of fatty acid metabolism deficits are linked to the dystrophic state and include carnitine uptake deficiencies, mitochondrial defects, and alterations to lipid composition [24] all suggesting that dystrophic muscle has a reduced fatty acid oxidation capacity. That we observe LC-AC accumulation—along with alterations in sphingolipid and sulfolipid metabolism—in SCs, suggests that impaired fatty acid metabolism may contribute to deficits in skeletal muscle regeneration. Indeed, several studies report that long-chain fatty acids and long-chain acylcarnitines interfere with satellite cell differentiation and provoke cell stress [35,36,37]. Whether these metabolite-driven defects act independently or in concert with signaling alterations (i.e., Notch [38], polarity proteins [12], heparin sulfate proteoglycans [39], and p38MAPK [23]) linked to dystrophic SCs is an important unanswered question.

Adipose tissue disequilibrium is a hallmark of DMD, with high levels of obesity observed in younger DMD patients, followed by dramatic adipose tissue loss [27,28]. Consistent with observations of altered adipose tissue equilibrium in patients with DMD, we noted significant adipose depletion in MDX mice. Accordingly, APCs exhibited reduced proliferation capacity, an observation that prompted us to perform metabolomic analyses on these cells. Similar to muscle SCs, we observed significant acylcarnitine accumulation suggesting that the dystrophic environment elicits conserved metabolic alterations in cell types outside of the skeletal muscle niche. Importantly, we noted that several LC-ACs were capable of slowing in vitro APC expansion. While our data do not prove that LC-ACs are the primary drivers of APC defects, they underscore the potential importance of dystrophy-associated metabolites as possible contributors to muscle and non-muscle tissue dysfunction in MDX mice. 

## 4. Conclusions

Using high content metabolomic analyses and the MDX mouse model of Duchenne muscular dystrophy, we demonstrate substantial metabolic alterations in dystrophic serum, muscle SCs, and APCs. We also acknowledge several study limitations and highlight corresponding opportunities for future work. First, our nontargeted analyses, while ideal for assessing the broader metabolite landscape, are not ideal for quantitative assessments of specific metabolite classes. To that end, targeted metabolite profiling would be important to perform in the future, especially before rigorously pursuing individual metabolites as putative drivers of tissue dysfunction in DMD. Second, our analysis only captured a single time-point along a continuum of disease progression. While we do observe notable differences at this time-point, a more extensive analysis of tissue-specific metabolic alterations over time would greatly improve our understanding of disease etiology. Third, we acknowledge that ectopic treatment of primary cells with acylcarnitines (or any metabolite) is not an accurate representation of normal or disease-associated physiology, but rather a proof-of-concept, preliminary study demonstrating the potentially growth-suppressing capabilities of long-chain acylcarnitine accumulation. Despite these limitations, this exploratory study lays the groundwork for future studies aiming to query metabolic deficiencies in progenitor cells in the context of disease.

## 5. Methods 

### 5.1. Satellite Cell Isolation

Satellite cells (SCs) were isolated from hind limb muscles using Satellite Cell Isolation Kit (Miltenyi Biotec; Bergisch Gladbach, Germany) as previously described [40] with the following modifications. The muscle tissues were digested with 0.2% (*w*/*v*) collagenase II (Gibco; Waltham, MA, USA) in 37 °C for 90 min. After isolation of the cells using Satellite Cell Isolation Kit (Miltenyi Biotec; Bergisch Gladbach, Germany), the cells were further purified with anti-integrin α-7 MicroBeads (Miltenyi Biotec; Bergisch Gladbach, Germany) according to the manufacturer’s protocol. 

### 5.2. Adipose Progenitor Cell Isolation

Epididymal, inguinal, and axillary fat pads were dissected immediately after mice were sacrificed. The fat pads were incubated in Hank’s Balanced Salt Solution (Gibco) containing 3% (*w*/*v*) bovine serum albumin (Gold Biotechnology; St. Louis, MO, USA) for 15 min at room temperature, followed by centrifugation at 200× g for 7 min. The fat pads were digested with 0.1% (*w*/*v*) collagenase II (Gibco; Waltham, MA, USA) in 37 °C for 60 min followed by filtering through 70 µm cell strainer. Adipose progenitor cells (APCs) were then enriched using Adipose Tissue Progenitor Isolation Kit (Miltenyi Biotec; Bergisch Gladbach, Germany) according to the manufacturer’s protocol. 

### 5.3. Nontargeted Metabolomics

Four biological replicates of serum, SCs, and APCs were prepared from Dmdmdx-4Cv (MDX) mice and wildtype (WT) control mice as follows. Blood was collected by submandibular bleeding with a lancet, as previously described [41]. After 20 to 30 min at room temperature, the collected blood was centrifuged at 1500× g for 15 min at 4 °C. Then, serum was collected by taking the resulting supernatant. SCs and AP Cs were isolated as described above. Approximately 100,000 cells (SCs) and 150,000 cells (APCs) were centrifuged at 400× g for 10 min at 4 °C, and the cell pellets were frozen immediately in liquid nitrogen. Serum, SCs, and APCs were submitted to Metabolomics Core at Mayo Clinic (Rochester, MN, USA) for nontargeted metabolomics profiling by liquid chromatography–mass spectrometry (LC–MS) using 6550 iFunnel Quadrupole Time of Flight (Q-TOF) mass spectrometer (Agilent; Santa Clara, CA, USA) coupled with an ultra-high pressure liquid chromatography (Agilent; Santa Clara, CA, USA) as previously described [42,43,44] with the following minor modifications. The samples were lysed in PBS and deproteinized with 1:1 acetonitrile/methanol, kept in ice with intermittent vortexing for 30 min, followed by centrifugation at 18,000× g. Three microliters of 13C6-phenylalanine (250 ng/µL) was added prior to deproteinization as internal standards. Mass spectrometer was operated in positive and negative electrospray ionization conditions using a scan range of 100 to 1700 *m*/*z*. Metabolite separation was performed using hydrophilic interaction column (HILIC) (ethylene-bridged hybrid 2.1 × 150 mm, 1.7 mm; Waters; Milford, MA, USA) and reversed-phase C18 column (high-strength silica 2.1 × 150 mm, 1.8 mm; Waters; Milford, MA, USA). The run times for HILIC and for C18 column were 18 min and 27 min, respectively. Putative identification of each metabolite was done based on accurate mass (*m*/*z*) against the Metlin database using a detection window of 7 ppm or less, as previously described [43].

### 5.4. Statistical Analysis and Metabolic Pathway Analysis

*T*-test was applied on intensities of metabolites in each type of sample to compare MDX mice and WT control mice. Metabolites with fold change greater than 1.5 fold and *p*-value lower than 0.05 were called significantly different metabolites. Principal component analysis (PCA) and hierarchical clustering were performed using TIBCO Spotfire Analyst 7.11.1 (TIBCO Software Inc.; Palo Alto, CA, USA). Clusters in hierarchical clustering were formed by complete linkage method with Euclidean distance. Metabolic pathway analysis was performed on significantly different metabolites using MetaboAnalyst [45] based on *Mus musculus* metabolic pathways. Since only a small fraction of the differentially abundant metabolites were able to be utilized by MetaboAnalyst for pathway analyses, less stringent *p*-values, as opposed to adjusted *p*-values, were used in these comparisons.

### 5.5. Animals and Imaging

All animal protocols were reviewed and approved by the Institutional Animal Care and Use Committee at Mayo Clinic (Rochester, MN, USA) (A3291-01). Four to six month old Dmd^mdx-4Cv^ (MDX) mice [46] were used for mice with muscular dystrophy phenotypes. C57BL/6J mice (Jackson Labs; Bar Harbor, ME, USA) were used for WT controls. Animal imaging was performed by dual energy X-ray absorptiometry (DEXA) scanning (Lunar PIXImus; Madison, WI, USA) on a restrained mouse anesthetized by ketamine/xylazine. Total amount of lean and fat was determined with the manufacturer’s software.

### 5.6. Cell Culture and Analysis

For proliferation experiments, equal numbers of WT or MDX APCs from a single mouse were plated in several wells of a 96 well plate. The cells were plated with growth media: DMEM containing 10% Fetal Bovine Serum (FBS), 1% penicillin-streptomycin (10,000 U/mL), and 0.01 µg/mL FGF2. Growth media was replaced every three days. To observe proliferation of WT APCs with and without carnitines, cells were first plated in growth media. After three days cells were change to growth media with vehicle control or growth media with a carnitine (propionyl-l-carnitine, butyryl-l-carnitine, octanoyl-l-carnitine, palmitoyl-l-carnitine, or stearoyl-l-carnitine from Sigma-Aldrich; St. Louis, MO, USA). Every three days thereafter the media was replaced. To measure proliferation, four images from each well were captured every four hours with an IncuCyte ZOOM (Essen Biosciences, Ann Arbor, MI, USA). The average confluence (cell occupying area) of all replicates was calculated and plotted over time using the IncuCyte ZOOM 2016B.

## Supplementary Materials

The following are available online at http://www.mdpi.com/2218-1989/8/4/61/s1, Figure S1: Flow cytometry analysis of SC marker integrin α-7 staining of column-isolated SCs. Figure S2: Metabolic pathways commonly identified in SCs, serum, and APCs. Figure S3: Flow cytometry analysis of APC marker Sca1 staining of column-isolated APCs. Figure S4: A bar graph quantifying the relative abundance of SC carnitine species. Table S1: SC differentially expressed metabolites, Table S2: Serum differentially expressed metabolites, Table S3: APC differentially expressed metabolites.
