# Supplementary material for: Metabolomic Analyses Reveal Extensive Progenitor Cell Deficiencies in a Mouse Model of Duchenne Muscular Dystrophy
Source: Metabolites. 2018 Oct 3;8(4):61. doi: 10.3390/metabo8040061 (PMC6315702; doi:10.3390/metabo8040061)
Supplement: Supplementary file 1 [file metabolites-08-00061-s001.pdf]

SUPPLEMENTARY FIGURES

Metabolomic analyses reveal extensive progenitor cell deficiencies in a mouse model of Duchenne muscular dystrophy

Josiane Joseph[1+], Dong Seong Cho[1+], Jason D Doles[1*]

[1]Department of Biochemistry and Molecular Biology, Mayo Clinic, Rochester, Minnesota, 55905 USA.

Author emails: Josiane Joseph (joseph.josiane@mayo.edu), Dong Seong Cho (cho.dong@mayo.edu), Jason D Doles (doles.jason@mayo.edu)

+Equal contribution

*Corresponding Author:
Jason D Doles
Department of Biochemistry and Molecular Biology
Mayo Clinic
200 First St SW
Guggenheim 16-11A1
Rochester, MN 55905
Tel: (507) 284-9372
Fax: (507) 284-3383
E-mail: Doles.Jason@mayo.edu

Supplementary Figure S1.

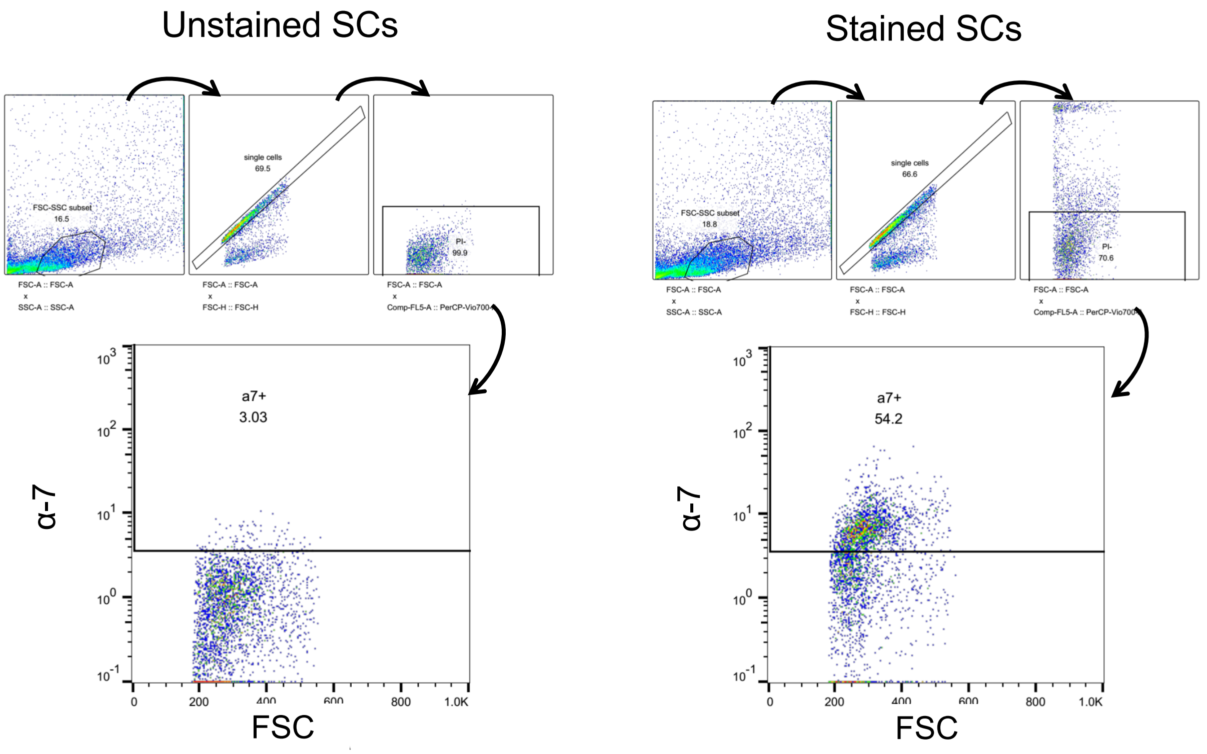

Supplementary Figure S1: Flow cytometry analysis of SC marker integrin α-7 staining of column-isolated SCs. Shown is the gating strategy employed to identify SCs.

Supplementary Figure S2.

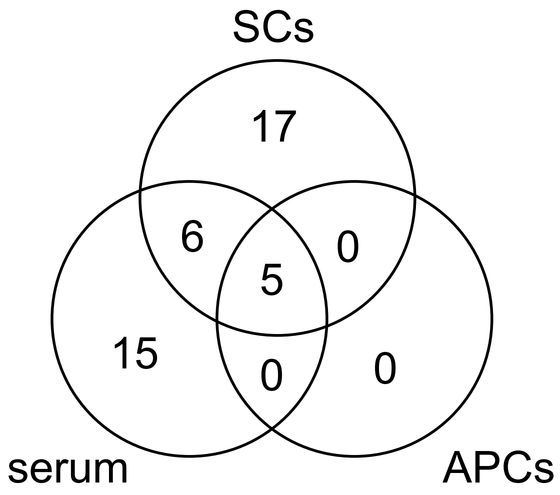

Common in

- SCs, serum, and APCs:
  - purine metabolism; pantothenate and CoA biosynthesis; aminoacyl-tRNA biosynthesis; valine, leucine and isoleucine biosynthesis; valine, leucine and isoleucine degradation
- SCs and serum:
  - citrate cycle (TCA cycle); arginine and proline metabolism; lysine degradation; glycolysis or gluconeogenesis; biosynthesis of unsaturated fatty acids; linoleic acid metabolism

Supplementary Figure S2: Metabolic pathways commonly identified in SCs, serum, and APCs. Based on Metaboanalyst pathway enrichment analysis.

Supplementary Figure S3.

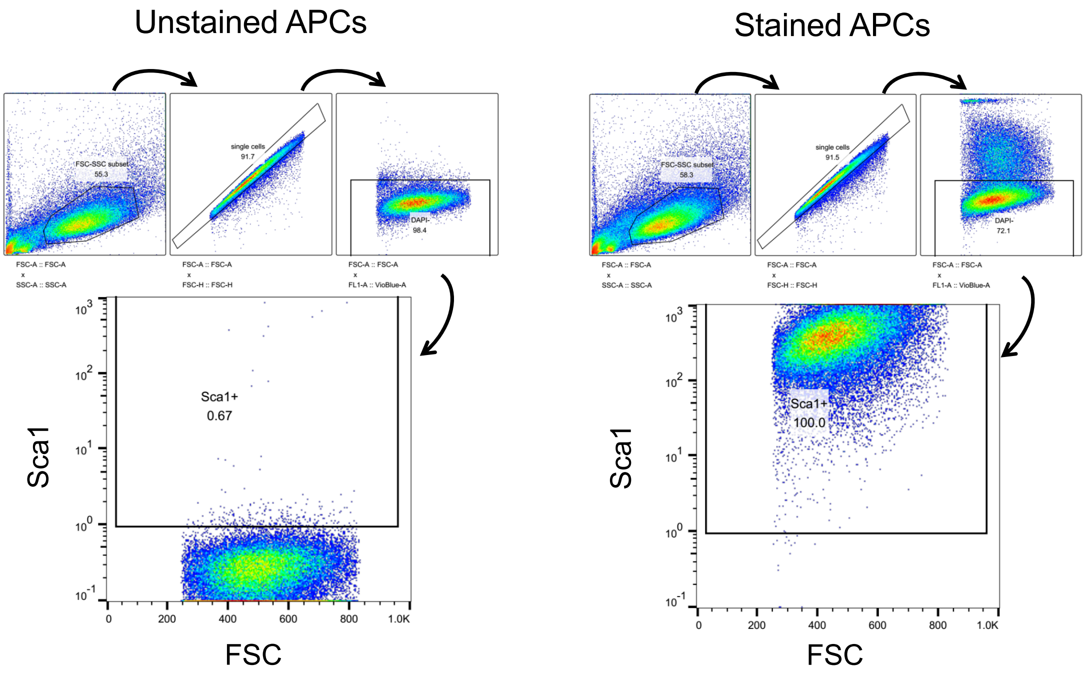

Supplementary Figure S3: Flow cytometry analysis of APC marker Sca1 staining of column-isolated APCs. Shown is the gating strategy employed to identify APCs.

Supplementary Figure S4.

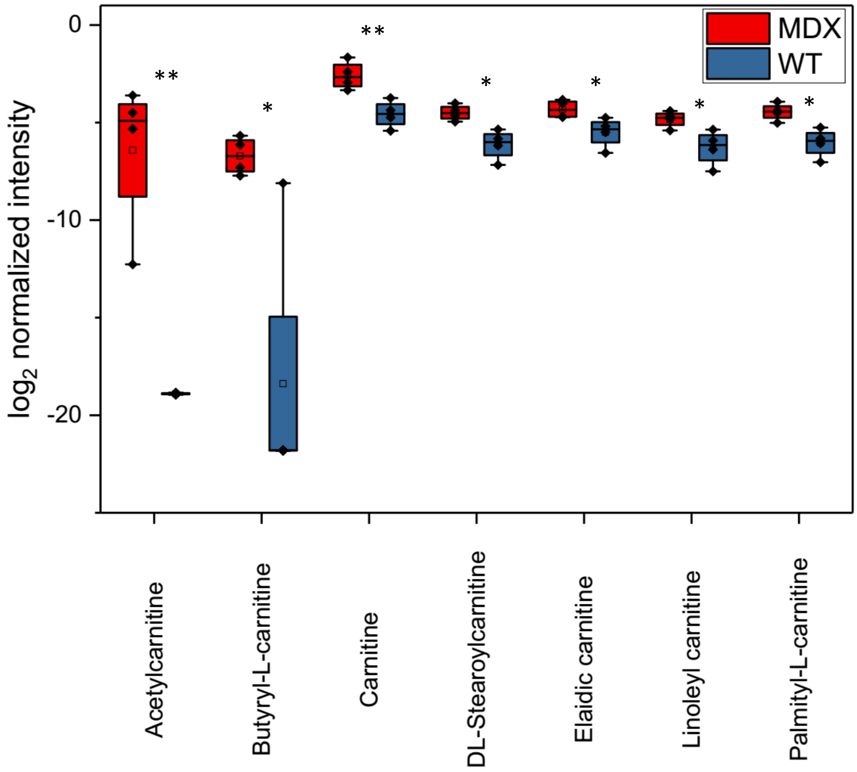

Supplementary Figure S4: A bar graph quantifying the relative abundance of SC carnitine species. *p<0.05, **p<0.01. n=4 mice in each experimental group.
